# Evaluating the Effects of Climate Change on Spatial Aggregation of Giant Pandas and Sympatric Species in a Mountainous Landscape

**DOI:** 10.3390/ani11113332

**Published:** 2021-11-22

**Authors:** Naxun Zhao, Ximing Zhang, Guoyu Shan, Xinping Ye

**Affiliations:** 1 Changqing Teaching & Research Base of Ecology, Shaanxi Normal University, Xi’an 710119, China; zhnx_fp@163.com (N.Z.); ximing2000@163.com (X.Z.); fpyxp@163.com (G.S.); 2 Administration of Shaanxi Changqing National Nature Reserve, Hanzhong 723000, China; 3 College of Life Sciences, Shaanxi Normal University, Xi’an 710119, China

**Keywords:** niche similarity, interspecific competition, spatial overlapping, giant pandas, sympatric species, climate change

## Abstract

**Simple Summary:**

Climate change has been regarded as one of the major threats to biodiversity by altering habitats and food sources for wildlife as well as the function of ecosystems. The giant panda is an endangered endemic species in China and a flagship species of the world’s biodiversity conservation. Previous studies mostly focused on the effect of climate change on the giant panda itself. Few studies have addressed potential niche overlapping and interspecific competition between giant pandas and sympatric competitive species under future climate change. By assessing the spatial overlapping between giant pandas and sympatric competitive animals changes under future climate conditions in the Qinling Mountains, we found that the distribution areas of giant pandas and sympatric species would decrease remarkably under future climate changes. The shifting of the spatial aggregation between giant pandas and sympatric species vary under different climate change scenarios. New protected areas may need to be planned in order to maintain suitable habitats able to promote the survival of the species to climate changes.

**Abstract:**

Understanding how climate change alters the spatial aggregation of sympatric species is important for biodiversity conservation. Previous studies usually focused on spatial shifting of species but paid little attention to changes in interspecific competitions under climate change. In this study, we evaluated the potential effects of climate change on the spatial aggregation of giant pandas (*Ailuropoda melanoleuca*) and three sympatric competitive species (i.e., black bears (*Ursus thibetanus*), golden takins (*Budorcas taxicolor*), and wild boars (*Sus scrofa*)) in the Qinling Mountains, China. We employed an ensemble species distribution modeling (SDM) approach to map the current spatial distributions of giant pandas and sympatric animals and projected them to future climate scenarios in 2050s and 2070s. We then examined the range overlapping and niche similarities of these species under different climate change scenarios. The results showed that the distribution areas of giant pandas and sympatric species would decrease remarkably under future climate changes. The shifting directions of the overlapping between giant pandas and sympatric species vary under different climate change scenarios. In conclusion, future climate change greatly shapes the spatial overlapping pattern of giant pandas and sympatric species in the Qinling Mountains, while interspecific competition would be intensified under both mild and worst-case climate change scenarios.

## 1. Introduction

Climate change can alter the spatial distributions of species, which may result in the changes in interspecies relationships [1]. Interspecific competition will arise from the spatial aggregation of two or more species in a limited space [1]. This competition may result in long-term effects on population dynamics or significant shifts in resource utilization to avoid overlapping. The maintenance of populations is subject to resources availability, viable populations, competition, and climate impacts in a changing environment [2]. Some studies have confirmed that both competition [3,4,5] and climate [6,7,8] are important factors driving species distribution. Climate change has influenced the distribution of wildlife and altered the habitat structure and function of many species since the Last Glacial Maximum (LGM; 24,000–18,000 years ago) [9,10,11]. An increasing number of species are responding to the changing climate by expanding or contracting their distribution ranges, which will continue as the climate warms [11,12,13].

Although few mammals are known to be directly affected by climate change, the intensity of interspecific competition may be affected by the changes of habitats and food sources under climate change [14]. A number of ecological studies have been performed on the topic of interspecific competition and climate change [15,16]. For example, interspecific competition in mixed forests under climate change has been explored [17]. Stenseth et al. [18] investigated the competitive interaction between blue tits and great tits under climate change, and found that climate change can, but does not always, generate local differences in the equilibrium conditions of spatially structured species assemblages. Milazzo et al. [19] and Braz et al. [20] highlighted that the consideration of species interactions is important for predictive modeling of responses to warming. However, little is known about the potential impact of climate change on the competitive patterns of multiple mammalian species. Understanding how the intensity of interspecific interactions responds to climate change would great help the conservation and management of species in a changing environment.

The giant panda (*Ailuropoda melanoleuca*) is an endangered endemic species in China and is a flagship species of the world’s biodiversity conservation. Research on giant pandas has attracted domestic and foreign scholars’ attention [21,22,23]. Some of these studies assessed the influence of climate change on the giant panda in the Qinling Mountains and found that the area of suitable habitat for giant pandas was projected to decrease [9,24,25,26]. The studies conducted in the Daxiangling and Qionglai Mountains indicated that suitable habitat loss will increase significantly under climate change as limited by the availability of bamboo and forest [27,28]. Shen et al. [29] combined long-term data on giant pandas with climate change scenarios and found that 11.4% of the remaining habitat fragments would be smaller than the extinction threshold area as the extent of fragmentation increases nearly fourfold. In the Qinling Mountains, black bears (*Ursus thibetanus*), golden takins (*Budorcas taxicolor*), and wild boars (*Sus scrofa*) are the main food competitors of giant pandas by eating bamboo shoots, which is an important energy source for nutritional recovery of giant pandas after the winter season [30]. However, previous studies mostly focused on the response of the giant panda itself to climate change, the interactions between giant pandas and sympatric large-bodied herbivores have been rarely examined [31,32,33]. There has been none on potential shifting of niche overlapping and interspecific competition between giant pandas and sympatric competitive species under climate change in the Qinling Mountains.

In this study, we predicted the current and future distributions of giant pandas and three sympatric species in the Qinling Mountains using species distribution models (SDMs), and analyzed niche similarities and potential interspecific competition under climate change scenarios. We evaluated how the degrees of niche similarity and spatial overlapping patterns of giant pandas and sympatric competitive animals change under future climate conditions, and predicted possible feeding pressures on giant pandas arising from interspecific competition in the future. The results will enrich our knowledge of the potential impacts of climate changes on giant pandas and have strong implications for the biodiversity conservation and management in the Qinling Mountains of China.

## 2. Materials and Methods

### 2.1. Study Area and Selected Species

**Figure 1:**
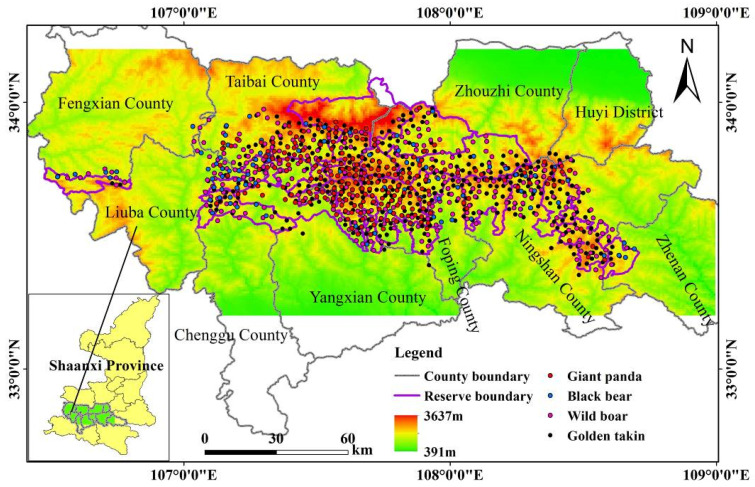
Location and elevation gradient of the study area in the Qinling Mountains in Shaanxi Province, China. The species occurrence points used in the study are displayed as points with different colors.

The study area (33°12′–34°12′ N, 106°24′–109° E) is located in the Qinling Mountains in Shaanxi Province (Figure 1), the natural boundary between northern and southern China and the northernmost mountain range that wild giant pandas inhabit (State Forestry Administration 2015). The study area is mostly covered by evergreen broadleaved and mixed deciduous broadleaved forests in the low- and mid-elevation regions and coniferous forests in the higher elevational bands (State Forestry Administration 2015). Bamboo forests (e.g., *Fargesia qinlingensis* and *Bashania fargesii*) are the dominant understory vegetation and nourish a large number of giant pandas. It is estimated that approximately 350 wild giant pandas are present in the study area, most of which live in Foping County, Yangxian County, and Taibai County [24,34]. Three sympatric mammals compete with giant pandas for bamboo resources: golden takins, wild boars, and black bears. According to a previous survey, over 4000 golden takins and 200 black bears live in the study area (State Forestry Administration 2015).

### 2.2. Species Occurrence Data

Original giant panda occurrence records were collected from the Third National Giant Panda Survey dataset, and the records of golden takins, black bears, and wild boars were obtained from the dataset of the sympatric species investigation performed during the giant panda survey. To reduce the effect of spatial autocorrelation, the original occurrence data were spatially thinned by using a distance of 2 km in ArcGIS 10.2 (ESRI, 2013). The resulting data contained 221 occurrence samples for giant pandas, 316 for wild boars, 445 for golden takins, and 96 for black bears, as shown in Figure 1. These data were used as input presence data for the SDMs of each species.

### 2.3. Environmental Variables

We firstly considered 22 environmental variables potentially affecting the distribution of the species, including 19 bioclimatic variables, elevation, slope, and aspect, based on previous research [9,26]. Climate data containing 19 bioclimatic variables were derived from WorldClim 1.4 (www.worldclim.org (accessed on 3 March 2019)) at 30 arc-second resolution (~1 km) for contemporary climatic conditions (1960–1990; hereafter ‘current’) as well as future climatic scenarios of 2041–2060 (hereafter ‘2050s’) and 2061–2080 (hereafter ‘2070s’). For future climate data, we selected two global climate models (GCMs) that were developed for the Coupled Model Intercomparison Project, phase 5 (CMIP5: BCC-CSM1-1 and MRI-CGCM3), which have been commonly used in previous climate modeling in the study area [35,36]. For each GCM, we chose two different representative concentration pathway (RCP) scenarios, RCP4.5, an optimistic scenario where greenhouse gas (GHG) emissions peak around 2040 and then decline, resulting in 4.5 W/m^2^ radiative forcing by 2100; and RCP8.5, a pessimistic scenario where emissions continue to rise throughout the 21st century, resulting in 8.5 W/m^2^ radiative forcing in the year 2100 [5,35,37,38,39]. Three topographical variables, i.e., elevation, slope, and aspect, were also derived from a digital elevation model at 30 arc-second resolution from the WorldClim database.

To identify a subset of environmental variables with minimal multicollinearity, we calculated the pairwise Pearson correlation coefficients for all 22 environmental variables. For a pair of variables with a correlation coefficient |r| > 0.7, we tested all environmental variables in a pairwise way, and retained the variable with the lowest variance inflation factor (VIF) in each pair of variables [25,40,41,42]. Finally, six variables were used for constructing the SDMs: isothermality (Bio03), annual precipitation (Bio12), precipitation seasonality (Bio15), elevation, slope, and aspect.

### 2.4. Species Distribution Modeling

We built the SDMs for each species under the current climate conditions and then projected them into the 2050s and 2070s scenarios via an ensemble modeling approach provided by the R package ‘biomod2′ [43]. Ensemble modeling is the process of running two or more related but different analytical models and then synthesizing the results into a single score in order to improve the accuracy of predictive analytics [43]. We used nine modeling techniques available in the ‘biomod2′ package for ensemble modeling: generalized linear models (GLMs), generalized boosted models (GBMs), classification tree analysis (CTA), artificial neural networks (ANNs), surface range envelopes (SREs), flexible discriminant analysis (FDA), multivariate adaptive regression splines (MARS), random forest (RF) and maximum entropy (MAXENT) [44]. As the absence of species was unavailable, we generated 10,000 pseudo-absences within the study area [45,46,47].

We used a random subset of 70% of the data to calibrate the model and the remaining 30% to evaluate it. Model performance was evaluated by the following measures: the area under the receiver operating characteristic (ROC) curve (AUC), the true skill statistic (TSS), and the kappa coefficient [48,49]. The AUC value is generally between 0.5 and 1, where a value higher than 0.7 is considered good model performance [50,51]. The TSS is a measure of agreement that accounts for both the sensitivity and specificity of the model [52,53]. The final distribution of each species was ensembled as the weighted mean of the nine modeling algorithms by the TSS scores (i.e., better a model is, more importance it has in the ensemble).

### 2.5. Spatial Analysis

To investigate how future climate change would affect spatially overlapping areas of giant pandas and their sympatric competitors, we performed a series of analyses in ArcGIS 10.2. Firstly, we refined the current and future species distributions by using land cover layers and classified habitats into binary data by the maximum training sensitivity plus the specificity logistic threshold [54,55]. We then calculated the binary layers of giant pandas and black bears (followed by golden takins and wild boars) by a raster calculator to generate habitat overlap maps. Finally, using the SDMtoolbox 2.2 package in ArcGIS 10.2 (http://www.sdmtoolbox.org (accessed on 10 January 2018)), we compared the distribution changes for each species between the current and future (the 2050s and 2070s) and, obtained the centroid changes of the species to depict the magnitude and direction of change based on centers of the species ranges.

### 2.6. Ecological Niche Similarity Analysis

The degrees of niche similarity between giant pandas and their sympatric competitors were calculated by using ENMTools 1.3 (http://purl.oclc.org/enmtools (accessed on 10 January 2018)). This software measures niche similarity using three statistics: Schoener’s D [56], I statistic [57] and relative rank (RR) [58]. In our study, D and I statistics were selected, which are obtained by comparing the normalized habitat suitability for each grid cell of the study area form SDMs [59]. Both metrics range from 0 to 1, in which the value of 0 indicates species-predicted environmental tolerances do not overlap and a value of 1 indicates that all the grid cells are estimated to be equally suitable for both species [59].

## 3. Results

### 3.1. Model Performance

The ensemble models of giant pandas, golden takins, and wild boars generally showed good predictive performance (Table 1). The ensemble model of black bears performed relatively poor, partially due to the small sample size (Table 1).

### 3.2. Suitable Habitat Change between the Current and Future Conditions

Compared with the current scenario, the distribution range of giant pandas will expand under the RCP4.5 emission scenario, and the suitable habitat area of giant pandas will increase by 36.61% and 25.74% by 2050s and 2070s, respectively (Table 2). The distribution range of black bears, golden takins and wild boars will contract under future climate change scenarios. Under the RCP8.5 emission scenario, the area of suitable habitats for black bears and wild boars will decrease by more than 80% by 2070 compared with the current area (Table 2).

### 3.3. Niche Similarity Analysis

The degrees of niche similarity between giant pandas and their sympatric competitors are shown in Table 3. Under current climate, giant pandas and golden takins had the highest niche similarity (*D* = 0.8454, *I* = 0.9697), while a lower level of niche similarity was observed between giant pandas and wild boars (*D* = 0.6976 and *I* = 0.9272). The lowest level of niche similarity was obtained between giant pandas and black bears (*D* = 0.6854, *I* = 0.9190). The highest niche similarity was still between giant pandas and golden takins under future climate change scenarios.

### 3.4. Analysis of Overlapping Area Changes

**Figure 2:**
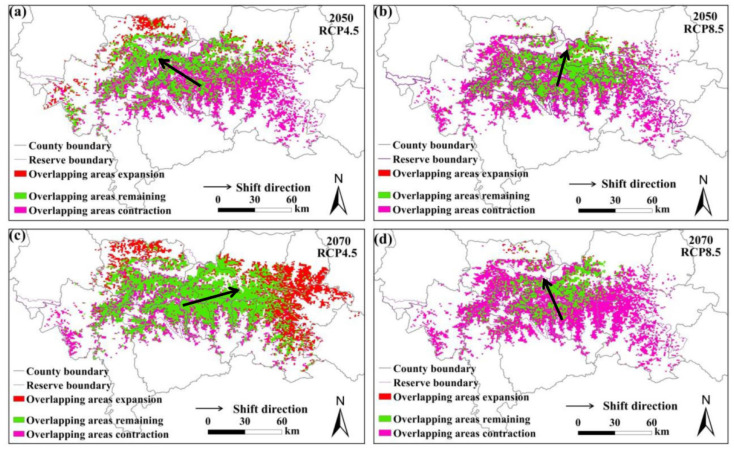
Overlapping suitable habitat for giant pandas and black bears under different climate change scenarios. (**a**,**b**) represent the changes under the RCP4.5 and RCP8.5 scenarios in 2050s, respectively; (**c**,**d**) represent the changes under the RCP4.5 and RCP8.5 scenarios in 2070s, respectively.

**Figure 3:**
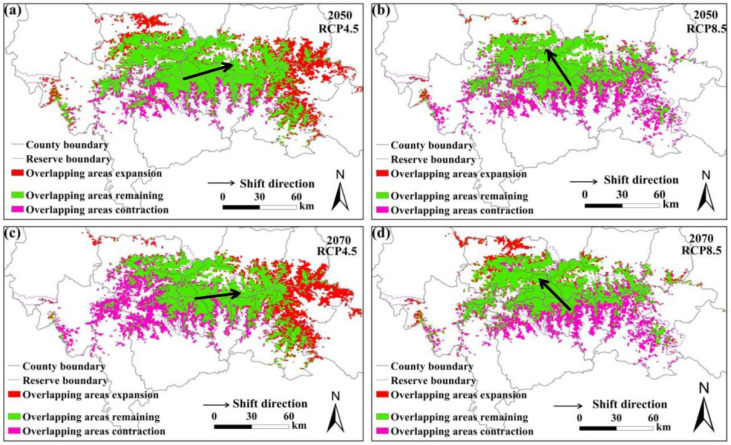
Overlapping suitable habitats of giant pandas and golden takins under different climate change scenarios. (**a**,**b**) represent the changes under the RCP4.5 and RCP8.5 scenarios in 2050s, respectively; (**c**,**d**) represent the changes under the RCP4.5 and RCP8.5 scenarios in 2070s, respectively.

**Figure 4:**
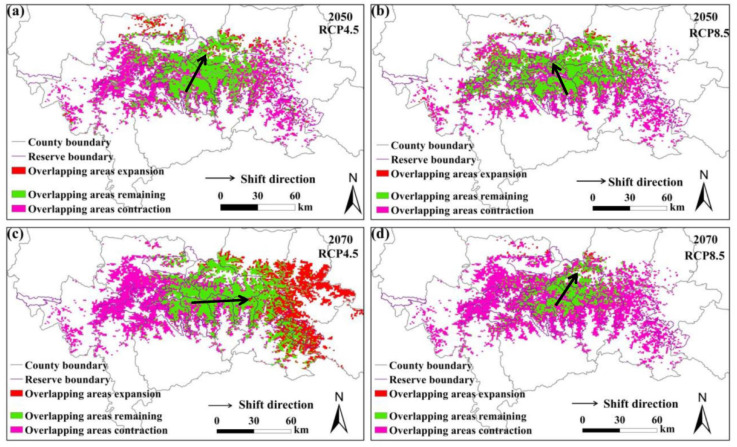
Overlapping suitable habitats of giant pandas and wild boars under different climate change scenarios. (**a**,**b**) represent the changes under the RCP4.5 and RCP8.5 scenarios in 2050s, respectively; (**c**,**d**) represent the changes under the RCP4.5 and RCP8.5 scenarios in 2070s, respectively.

The predicted overlapping of the suitable habitats for giant pandas and black bears, golden takins, and wild boars is shown in Figure 2, Figure 3 and Figure 4, respectively. Compared with the current habitats, the overlapping habitats between giant pandas and competitive animals will generally decrease under future climate scenarios (Table 4). Under the RCP4.5 emission scenario, the overlapping areas for giant pandas and black bears will expand in the eastern Qinling Mountains by 2070s (Figure 2). The centroid shifting trends of overlapping areas between giant pandas and black bears will vary under different climate change scenarios. The overlapping areas for giant pandas and golden takins will expand mainly to the east of the Qinling Mountains under the RCP4.5 emission scenario, whereas the overlapping area centroids will shift to the northwest under the RCP8.5 emission scenario (Figure 3). The shifting directions of centroids of overlapping areas between giant pandas and wild boars varied under different climate change scenarios (Figure 4).

**Figure 5:**
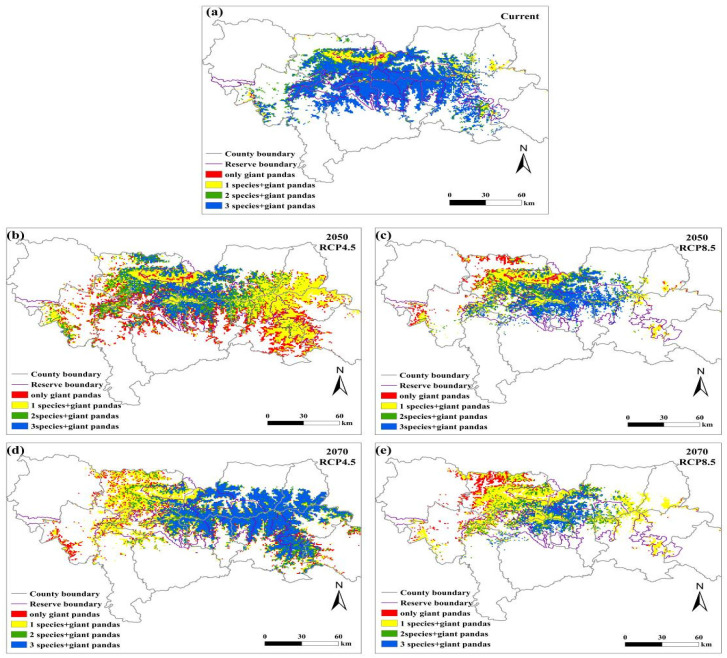
Overlapping distributions of the suitable habitats for giant pandas, black bears, golden takins, and wild boars in the Qinling Mountains under the (**a**) current climate, (**b**) RCP4.5 scenario in 2050s, (**c**) RCP8.5 scenario in 2050s, (**d**) RCP4.5 scenario in 2070s, and (**e**) RCP8.5 scenario in 2070s.

The number of species overlapping with giant pandas will decrease in the central Qinling Mountains under future climate change scenarios, indicating that potential competition for food resources faced by giant pandas in the central part of the Qinling Mountains is likely to decrease (Figure 5). Interestingly, the competitive pressure on giant pandas in the eastern Qinling Mountains may increase by 2070s under the RCP4.5 emission scenario.

## 4. Discussion

The study evaluated the potential impact of future climate change on the competitive patterns of giant pandas and sympatric competitors in the Qinling Mountains of China. Current habitat distribution pattern of giant pandas is consistent with the results of the Fourth National Giant Panda Survey in Shaanxi Province, which to some extent confirms the reliability of our model. In this study, the simulated habitat area for giant pandas was greater than the results of the report on the Fourth National Giant Panda Survey [60] and those of Songer et al. [36]. This difference may be caused by differences in environmental factors, selected models and habitat suitability classification methods and thresholds, or the model may not be able to simulate the real habitat conditions completely. Our results show that new areas will become suitable for the species in the future. However, most of these areas are far from the current geographical distribution and are beyond the scope of existing protected areas. Therefore, new protected areas may need planning in order to create suitable habitats able to promote the survival of the species to climate changes.

The degree of niche similarities between giant pandas and the sympatric competitive species is high, which indicates that there are great overlaps in habitat use between giant pandas and sympatric competitors. This result is similar to the findings of other studies that showed no evidence of sympatric species restricting the distribution of giant pandas [30]. Although spatial avoidance is a strong indicator that interactions exist, not all competition between pandas and the other species would be reflected in spatial avoidance, partially due to the temporal separation from consuming same resources [30,61,62]. The results of the Fourth National Giant Panda Survey also showed that golden takins are the sympatric species giant pandas most likely to encounter, followed by wild boars and black bears [60]. Some studies have shown that golden takins are the most competitive species due to the high consumption of bamboo leaves, while black bears and wild boars are thought to alter giant panda habitat selection by foraging for bamboo shoots [30,63,64]. As with the studies of Milazzo et al. [19], Poloczanska et al. [14], and Stenseth et al. [18], our results suggest that the overlapping patterns of giant pandas and sympatric competitors and their overlapping area centroids will change significantly under future climate change, indicating that current protection network will face big challenges under future climate changes.

The Qinling Mountains are a hotpot of biodiversity in China. Numerous nature reserves have been built for giant pandas, crested ibis and other key protected species. The existing reserve system provides adequate protection for giant pandas. Giant pandas can be regarded as an umbrella species. While carrying out giant pandas’ protection, the ‘umbrella effect’ also protects other sympatric species [65,66,67]. However, not all these species can be fully protected. The fact is, current reserves were established solely for conserving giant pandas. In the context of biodiversity conservation, we need to monitor the dynamics of sympatric animals in addition to giant pandas so as to protect them under future climate changes in the Qinling Mountains.

Some uncertainties may influence the results of this study. Some models in the package “biomod2” require both presence and absence data, while others require presence data only. To ensure the operation of all models, the pseudo-absence data were generated in the “biomod2” package, which may affect model prediction [45,46,47]. Therefore, the model performance conclusion of this paper may be optimistic. The selection of SDMs has an overarching influence on the final results compared to the choice of GCMs and RCPs [68]. The premise of applying a climatic SDM is that climate factors are the main limiting factors of matter distribution [69]. Nevertheless, our results showed that current reserve network may fail to protect biodiversity adequately under future climate, which has important implications for multispecies management and regional biodiversity conservation under climate changes.

## 5. Conclusions

The patterns of spatial aggregation of giant pandas and sympatric species changed remarkably under future climate changes in the Qinling Mountains, China. The degree of niche similarity and spatial overlapping patterns between giant pandas and sympatric competitive animals will also be greatly shaped by the change of climate conditions. The shifting of the overlap between giant pandas and sympatric species varies under different climate change scenarios. New protected areas need to be established outside current reserve network so as to maintain biodiversity under future climate changes.

## Figures and Tables

**Table 1:** Performances of the SDMs for giant pandas and sympatric competitive species. TSS: true skill statistic; AUC: area under the receiver operating characteristic curve.

Species	Kappa	TSS	AUC
Giant pandas	0.631	0.769	0.937
Black bears	0.313	0.581	0.822
Golden takins	0.669	0.717	0.916
Wild boars	0.537	0.620	0.866

**Table 2:** Statistics on the areas of suitable habitats for giant pandas and sympatric competitive species under different climate scenarios.

Species	Current	2050s		2070s	
		RCP4.5	RCP8.5	RCP4.5	RCP8.5
Giant pandas	6566.31	8969.98	4603.31	8256.33	4916.34
Black bears	7947.02	2987.82	3350.23	6576.55	1529.78
Golden takins	6989.28	6548.60	4609.83	5484.65	5426.89
Wild boars	7957.27	2674.78	4681.57	4668.52	1475.74

**Table 3:** Niche similarity between giant pandas and sympatric competitive species under different climate scenarios.

Climate Scenarios	Black Bears		Golden Takins		Wild Boars	
	*D*	*I*	*D*	*I*	*D*	*I*
Current	0.6854	0.9190	0.8454	0.9697	0.6976	0.9272
2050-RCP4.5	0.7444	0.9392	0.8651	0.9829	0.8147	0.9676
2050-RCP8.5	0.6851	0.9163	0.8034	0.9609	0.6689	0.9131
2070-RCP4.5	0.7604	0.9491	0.8662	0.9818	0.8256	0.9690
2070-RCP8.5	0.7101	0.9251	0.8401	0.9694	0.6943	0.9225

**Table 4:** Statistics on overlapping area of suitable habitats between giant pandas and sympatric competitive species under different climate scenarios. Calculated as the proportion of suitable habitat area for giant pandas (%).

Climate Scenarios	Black Bears	Golden Takins	Wild Boars
Current	86.30	88.64	86.16
2050-RCP4.5	30.97	71.37	29.11
2070-RCP4.5	72.19	65.41	55.72
2050-RCP8.5	48.73	77.66	56.06
2070-RCP8.5	27.82	87.02	25.43

## Data Availability

Not applicable.
